# An explorative analysis of the prognostic value of lactate dehydrogenase for survival and the chemotherapeutic response in patients with advanced triple-negative breast cancer

**DOI:** 10.18632/oncotarget.24246

**Published:** 2018-01-13

**Authors:** Zhenya Jia, Jian Zhang, Zhonghua Wang, Biyun Wang, Leiping Wang, Jun Cao, Zhonghua Tao, Xichun Hu

**Affiliations:** ^1^ Department of Medical Oncology, Fudan University Shanghai Cancer Center, Department of Oncology, Shanghai Medical College, Fudan University, Shanghai 200032, China; ^2^ Department of Oncology, The First Affiliated Hospital of Anhui Medical University, Hefei 230032, China

**Keywords:** triple-negative breast cancer, lactate dehydrogenase, metastasis, prognosis

## Abstract

Serum lactate dehydrogenase (LDH) level is predictive of prognosis in various malignancies. Nevertheless, the association between the prognosis of patients with advanced triple-negative breast cancer (TNBC) and LDH is not well understood. This explorative and retrospective study was conducted to clarify the issue. We found that abnormal baseline LDH levels (> 250 IU/L) were significantly associated with age (> 40 y vs. ≤ 40 y, OR: 0.383, *P* = 0.031) and number of metastatic sites (2 vs. 1, OR: 4.619, *P* = 0.006; ≥ 3 vs. 1, OR: 4.727, *P* = 0.002). The progression-free survival (PFS) of patients with post-treatment LDH higher than baseline (Group 1) was significantly shorter than that in patients with LDH decreased to normal (Group 3) and those with normal baseline and post-treatment LDH (Group 4) (Group 3 vs. Group 1, HR: 0.517, *P* = 0.038; Group 4 vs. Group 1, HR: 0.346, *P* < 0.001). Overall survival (OS) in patients with abnormal baseline LDH was significantly shorter than in patients with normal baseline LDH (abnormal vs. normal, HR: 2.073, *P* < 0.001). Patients whose post-treatment LDH decreased to normal had the most objective response (complete and partial responses) rate after first-line chemotherapy (Group 3 vs. Group 1, OR: 0.074, *P* < 0.001). In this exploratory analysis, baseline LDH levels associated with OS, while LDH changes after first-line chemotherapy associated with PFS and the chemotherapeutic response. These results show that LDH may have important prognostic value for the survival and chemotherapeutic response in patients with advanced TNBC.

## INTRODUCTION

Breast cancer is the second leading cause of cancer-related deaths in women and is the most common cancer in females, accounting for 23% of all cancer cases [1, 2]. Triple-negative breast cancer (TNBC) is a subtype characterized by the lack of estrogen receptor (ER), progesterone receptor (PR), and human epidermal growth factor receptor 2 (HER-2) expression, as confirmed by immunohistochemistry or fluorescent *in situ* hybridization. TNBC is associated with a poor prognosis due to its more aggressive behavior, higher recurrence, generation of more metastases, and fewer treatment options compared with other breast cancer subtypes [3]. Cytotoxic chemotherapy remains the mainstay of treatment for TNBC, and metastatic organ sites and disease-free interval (DFI) are thought to be prognostic factors of advanced disease [4].

Serum lactate dehydrogenase (LDH) converts pyruvate to lactate in the cytoplasm during glycolysis and is thought to be a marker of tissue injury, inflammation, hemolysis, and myocardial damage [5–7]. In addition, abnormally high serum levels of LDH are predictive of prognosis in various malignancies [8] and are associated with breast cancer patient survival [4, 9–10]. Nevertheless, the association between advanced TNBC and serum LDH is not well understood; thus, we conducted an explorative study to determine the potential relationship.

## RESULTS

**Figure 1:**
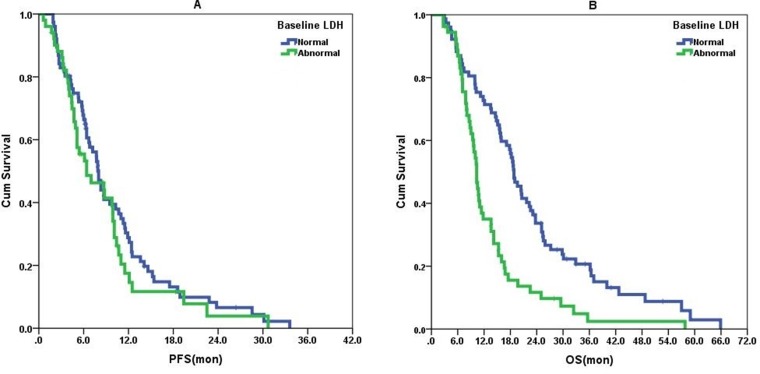
Baseline serum LDH level as a prognostic indicator of survival There was no difference in PFS between advanced TNBC patients with abnormal baseline serum LDH levels (*n* = 54) and those with normal baseline serum LDH levels (*n* = 77) in (**A**) (unadjusted HR: 1.237, 95% CI: 0.837–1.828, *P* = 0.284). Advanced TNBC patients with abnormal baseline serum LDH levels (*n* = 54) had significantly poorer OS than those with normal baseline levels (*n* = 77) in (**B**) (unadjusted HR: 2.192, 95% CI: 1.504–3.194, *P* < 0.001).

**Figure 2:**
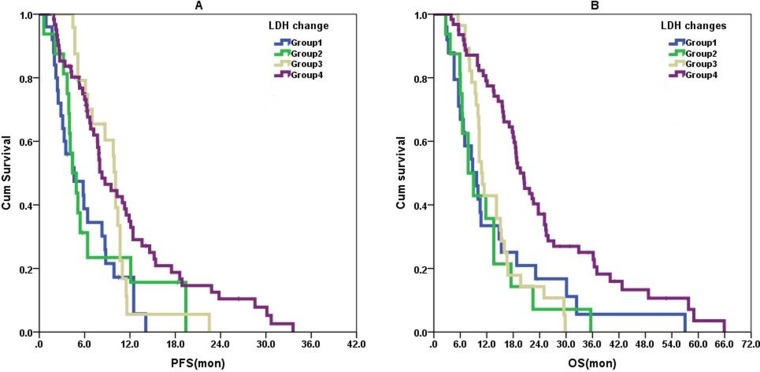
Serum LDH level changes as the prognostic variable in survival curves There was a significant difference in PFS among the four groups defined by the changes of serum LDH in (**A**) (*P* = 0.004) even if adjusted with other variables. However, the difference in OS among the four groups in (**B**) (*P* < 0.001) disappears when adjusted. Group 1: Abnormal and higher than baseline; Group 2: Did not return to normal; Group 3: Decreased to normal; Group 4: Both normal.

All patients received a platinum-based or taxane-based regimen as first-line treatment (Table 1). In our daily practice, the previously used drugs will not be reapplied in those patients experiencing relapse within 3 months after completing neo-adjuvant or adjuvant chemotherapies. Table 2 shows the association between baseline serum LDH levels and clinical characteristics. Age (> 40 y vs. ≤ 40 y, odds ratio [OR]: 0.383, 95% confidence interval [CI]: 0.160–0.918, *P* = 0.031) and number of metastatic sites (2 vs. 1, OR: 4.619, 95% CI: 1.558–13.694, *P* = 0.006; ≥ 3 vs. 1, OR: 4.727, 95% CI: 1.777–12.570, *P* = 0.002) independently associated with abnormally high baseline serum LDH levels above normal. Univariate analyses revealed that DFI, number of metastatic sites, chemotherapeutic response, and LDH changes were potential prognostic factors for PFS (Table 3), and DFI, number of metastatic sites, liver, skeletal and lymph node metastases, chemotherapeutic response, and baseline serum LDH and LDH changes were potential prognosis factors for OS (Table 4). PFS and OS of different groups are shown in Figures 1 and 2. Patients with abnormally high baseline serum LDH levels above normal had significantly shorter OS (unadjusted HR: 2.192, 95% CI: 1.504–3.194, *P* < 0.001) but no difference in PFS (unadjusted HR: 1.237, 95% CI: 0.837–1.828, *P* = 0.284) compared to those with normal baseline serum LDH levels (Figure 1).

**Table 1:** Baseline clinical characteristics

Characteristics		No. of patients (%)
Age	≤ 40 years	31 (23.7)
	> 40 years	100 (76.3)
Menopausal status	Pre-menopause	75 (57.3)
	Post-menopause	56 (42.7)
Number of metastatic sites	1	40 (30.5)
	2	32 (24.4)
	≥ 3	59 (45.1)
Liver metastases	Absent	94 (71.8)
	Present	37 (28.2)
Lung metastases	Absent	69 (51.8)
	Present	63 (48.1)
Skeletal metastases	Absent	83 (63.4)
	Present	48 (36.6)
Lymph node metastases	Absent	39 (29.8)
	Present	92 (70.2)
Disease-free survival	DFI > 12 months	70 (53.4)
	DFI ≤ 12 months	51 (38.9)
	Primary metastases	10 (7.6)
Chemotherapy response	Yes	77 (58.8)
	No	54 (41.2)
Baseline serum LDH	Normal	77 (58.8)
	Abnormal	54 (41.2)
LDH level Changes	Group 1: Abnormal and higher than baseline	25 (19.1)
	Group 2: Did not return to normal	16 (12.2)
	Group 3: Decreased to normal	28 (21.4)
	Group 4: Both normal	62 (47.3)

**Table 2:** The difference in baseline serum LDH status (normal vs abnormal) according to various baseline characteristics

Variables	Baseline serum LDH status			Logistic regression model	
	Normal (≤ 250 IU/L) *n* (%)	Abnormal (> 250 IU/L) *n* (%)	P^*^	OR (95%CI)	P^**^
Age			*0.009*		
≤ 40 years	12 (38.7%)	19 (61.3%)		Ref.	
> 40 years	65 (65.0%)	35 (35.0%)		0.383 (0.160–0.918)	*0.031*
Menopausal status			*0.068*		
Pre-menopause	39 (52.0%)	36 (48.0%)			
Post-menopause	38 (67.9%)	18 (32.1%)			
Number of metastatic sites			*0.001*		
1	33 (82.5%)	7 (17.5%)		Ref.	
2	16 (50.0%)	23 (50.0%)		4.619 (1.558–13.694)	*0.006*
≥ 3	28 (47.5%)	31 (52.5%)		4.727 (1.777–12.570)	*0.002*
Liver metastases			*0.139*		
Absent	59 (62.8%)	35 (37.2%)			
Present	18 (48.6%)	19 (51.4%)			
Lung metastases			*0.714*		
Absent	41 (60.3%)	27 (39.7%)			
Present	36 (57.1%)	27 (42.9%)			
Skeletal metastases			*0.008*		*0.137*
Absent	56 (67.5%)	27 (32.5%)			
Present	21 (43.8%)	27 (56.3%)			
Lymph node metastases			*0.027*		*0.563*
Absent	29 (74.4%)	10 (25.6%)			
Present	48 (52.2%)	44 (47.8%)			
Disease-free survival			*0.365*		
> 12 months	44 (62.9%)	26 (37.1%)			
≤ 12 months	29 (56.9%)	22 (43.1%)			
Primary metastatic	4 (40.0%)	6 (60.0%)			

^*^chi-squared test; ^**^Logistic regression model with multiple variables (age, menopausal status, number of metastatic sites, liver metastases, lung metastases, skeletal metastases, lymph node metastases, and disease-free survival)

**Table 3:** Univariate and multivariate analysis of prognostic factors in PFS of advanced TNBC patients

Variables	Univariate analysis		Multivariate analysis	
	Median PFS(months)	P^*^	HR(95%CI)	P^**^
Disease-free survival		*0.023*		
> 12 months	11.3		Ref.	
≤ 12 months	7.5		1.976 (1.300–3.003)	*0.001*
Primary metastatic	7.50		1.867 (0.831–4.194)	*0.130*
Number of metastatic sites		*0.012*		
1	12.2			
2	8.7			
≥ 3	7.8			
Chemotherapy response		*< 0.001*		
Yes	11.6		Ref.	
No	6.7		2.684 (1.787–4.032)	< *0.001*
LDH level Changes		*0.004*		
Group 1	6.0		Ref.	
Group 2	7.1		0.815 (0.412–1.612)	*0.557*
Group 3	9.4		0.517 (0.278–0.963)	*0.038*
Group 4	11.4		0.346 (0.204–0.587)	< *0.001*

^*^ Log-rank test; ^**^Cox regression model with multiple variables(disease-free survival, number of metastatic sites, chemotherapy response and LDH level Changes)

**Table 4:** Univariate and multivariate analysis of prognostic factors in OS of advanced TNBC patients

Variables	Univariate analysis		Multivariate analysis	
	Median OS(months)	P^*^	HR(95%CI)	P^**^
Disease-free survival		*0.002*		
> 12 months	23.2		Ref.	
≤ 12 months	14.7		1.888 (1.252–2.848)	0.002
Primary metastatic	13.6		1.571 (0.788–3.132)	0.119
Number of metastatic sites		*< 0.001*		
1	27.7		Ref.	
2	18.9		1.839 (1.805–3.118)	0.024
≥ 3	13.1		2.449 (1.548–3.874)	< 0.001
Liver metastases		*0.005*		
Absent	21.0			
Present	13.6			
Skeletal metastases		*0.004*		
Absent	21.5			
Present	14.3			
Lymph nodes metastases		*0.001*		
Absent	26.3			
Present	15.7			
Chemotherapy response		*0.019*		
Yes	21.3		Ref.	
No	15.4		1.754 (1.203–2.559)	0.004
Baseline LDH level		*< 0.001*		
Normal	22.8		Ref.	
Abnormal	13.4		2.073 (1.397–3.074)	< 0.001
LDH level Changes		*< 0.001*		
Group 1	13.9			
Group 2	11.8			
Group 3	14.2			
Group 4	24.9			

^*^Log-rank test; ^**^Cox regression model with multiple variables(disease-free survival, number of metastatic sites, liver metastases, lung metastases, skeletal metastases, lymph node metastases, chemotherapy response, baseline LDH level and LDH level Changes).

Using the Cox regression model, we found that the independent prognostic factors of PFS for advanced TNBC patients were DFI (≤ 12 mon vs. > 12 mon, HR: 1.976, 95% CI: 1.300–3.003, *P* = 0.001), chemotherapeutic response (no vs. yes, HR: 2.684, 95% CI: 1.787–4.032, *P* < 0.001), and LDH changes (Group 3 vs. Group 1, HR: 0.517, 95% CI: 0.278–0.963, *P* = 0.038; Group 4 vs. Group1, HR: 0.346, 95% CI: 0.204–0.587, *P* < 0.001). The difference in OS among the four groups defined by LDH changes in Figure 2 disappeared when adjusted with other variables identified by univariate analysis with *P* < 0.1 (DFI, number of metastatic sites, liver metastases, skeletal metastases, lymph nodes metastases, chemotherapy response, and baseline LDH level). Actually, only DFI (≤ 12 mon vs. > 12 mon, HR = 1.888, 95% CI: 1.252–2.848, *P* = 0.002), number of metastatic sites (2 vs. 1, 95% CI: 1.805–3.118, *P* = 0.024; ≥ 3 vs. 1, HR: 2.449, 95% CI: 1.548–3.874, *P* < 0.001), chemotherapeutic response (no vs. yes, HR: 1.754, 95% CI: 1.203–2.559, *P* = 0.004) and baseline LDH (high vs. normal, HR: 2.073, 95% CI: 1.397–3.074, *P* < 0.001) were independent prognostic factors of OS for advanced TNBC patients.

Associations between clinical characteristics and chemotherapeutic responses are summarized in Table 5. No response to chemotherapy (SD or PD) was significantly associated with the presence of liver metastases (present vs. absent, OR: 4.267, 95% CI: 1.703–10.692, *P* = 0.002) and LDH changes (Group 3 vs. Group 1, OR: 0.074, 95% CI: 0.018–0.311, *P <* 0.001) according to logistic regression. Patients with post-treatment serum LDH that decreased to normal had the greatest ORR. With regard to ORR, Group 1 patients did not show inferiority compared to groups 2 and 4. Liver metastases and post-treatment LDH that decreased to normal were independently associated with ORR according to logistic regression.

**Table 5:** The difference in clinical response (CR or PR vs SD or PD) according to various clinical characteristics

Variables	Clinical response			Logistic regression model	
	CR or PR n (%)	SD or PD n (%)	P^*^	OR (95%CI)	P^**^
Age			*0.745*		
≤ 40 years	19 (61.3%)	12 (38.7%)			
> 40 years	58 (58.0%)	42 (42.0%)			
Menopausal status			*0.976*		
Pre-menopause	44 (58.7%)	31 (41.3%)			
Post-menopause	33 (58.9%)	23 (41.1%)			
Number of metastatic sites			*0.413*		
1	26 (65.0%)	14 (35.0%)			
2	20 (62.5%)	12 (37.5%)			
≥ 3	31 (52.5%)	28 (47.5%)			
Liver metastases			*0.008*		
Absent	62 (66.0%)	32 (34.0%)		Ref.	
Present	15 (40.5%)	22 (59.5%)		4.267 (1.703–10.692)	*0.002*
Lung metastases			*0.077*		
Absent	35 (51.5%)	33 (48.5%)			
Present	42 (66.7%)	21 (33.3%)			
Skeletal metastases			*0.415*		
Absent	51 (61.4%)	32 (38.6%)			
Present	26 (54.2%)	27 (45.8%)			
Lymph node metastases			*0.420*		
Absent	25 (64.1%)	14 (35.9%)			
Present	52 (56.5%)	40 (43.5%)			
Disease-free survival			*0.365*		
> 12 months	26 (51.0%)	25 (49.0%)			
≤ 12 months	44 (62.9%)	26 (37.1%)			
Primary metastatic	7 (70.0%)	3 (30.0%)			
Baseline serum LDH			*0.415*		
Normal	43 (55.8%)	34 (44.2%)			
Abnormal	34 (63.0%)	20 (37.0%)			
LDH level Changes			*0.005*		
Group 1	10 (40.0%)	15 (60.0%)		Ref.	
Group 2	8 (50.0%)	8 (50.0%)		0.555 (0.145–2.121)	*0.390*
Group 3	24 (85.7%)	4 (14.3%)		0.074 (0.018–0.311)	*< 0.001*
Group 4	35 (56.5%)	27 (43.5%)		0.537 (0.201–1.437)	*0.216*

^*^chi-squared test; ^**^Logistic regression model with multiple variables (age, menopausal status, number of metastatic sites, liver metastases, lung metastases, skeletal metastases, lymph node metastases, disease-free survival, baseline serum LDH and LDH level Changes).

## DISCUSSION

Advanced TNBC is highly aggressive, with a median PFS of 3–7.7 months and a median OS of about 1 year [11–15]. Similarly, the PFS and OS in this study population were 7.9 months (95% CI: 6.3–9.5 months) and 15.1 months (95% CI: 13.2–17.0 months), respectively.

LDH is required for aerobic glycolysis and can reversibly catalyze conversion of pyruvate to lactate. Recently, serum LDH has been reported to be important in numerous malignances and is documented to be 1 of 5 risk factors in the International Prognostic Index for the diffuse large B-cell lymphoma [16]. Baseline serum LDH has also been included in TNM staging system of melanoma [17]. Furthermore, high serum LDH levels are associated with adverse outcomes in lung cancer [18], esophageal squamous cell carcinoma [19], gastric [20] and pancreatic cancer [21] and renal cell carcinoma [22]. A recent systematic review and meta-analysis identified 76 studies of various solid tumors in which higher LDH was associated with shorter OS (HR = 1.7, *P <* 0.00001) and shorter PFS (HR = 1.75, *P <* 0.00001) [23]. In addition, LDH is reported to be a promising predictor of effectiveness of targeted agents such as bevacizumab, vatalanib, and sorafenib [24–26].

Although multiple studies have demonstrated the prognostic value of LDH in various malignances, the underlying pathophysiological mechanism remains unclear. LDH is possibly translationally controlled by HIF-1 and myc, and thus is regulated by the key oncogenic processes such as the phosphatidylinositol 3-kinase/AKt/TORC1/HIF pathway or by myc overexpression [27–29]. There is a positive feedback loop between HIF and LDH, and each can stimulate the activation of the other [30]. Moreover, HIF overexpression can activate vascular endothelial growth factor-A [31], thereby linking glycolysis and LDH to angiogenesis and cancer progression [32].

Here, we exploratively studied the association between serum LDH and prognosis of advanced TNBC patients, and found that abnormal serum LDH levels were significantly associated with metastatic sites and younger age of cancer onset. Greater metastatic sites reflect heavier tumor burden and younger patients tend to have poorer prognosis as compared to older patients [33, 34], and our data indicated that LDH might negatively influence the progression of advanced TNBC. However, menopausal status, liver, lung, skeleton, lymph node metastases and DFI were shown to have no effect on baseline serum LDH. Few studies describe the prognostic value of serum LDH in breast cancer. Yamamoto's group[4] reported that abnormal serum LDH was associated with poorer survival among metastatic breast cancer subjects and Brown's group[10] confirmed that LDH was strongly correlated with survival in breast cancer patients with bone metastases. Moreover, Liu and colleague [9] reported that greater LDH predicted worse 5-year OS in non-metastatic stage II and III breast cancers. We also found significant associations between serum LDH level and prognosis of advanced TNBC patients. However, an independent prognostic value was only found with LDH changes between baseline and post-treatment for PFS and baseline LDH for OS. Patients with post-treatment serum LDH level that decreased to normal and patients who had both normal baseline and post-treatment serum LDH had significantly longer PFS than patients whose post-treatment serum LDH were abnormal and higher than baseline. In addition, patients with normal baseline serum LDH had significantly longer OS than patients with abnormal baseline serum LDH. As mentioned earlier, abnormal serum LDH might have a negative effect on the progression of advanced TNBC, and this may partially explain why patients with normal post-treatment serum LDH level had better survival outcomes.

Decreases in LDH may reflect cessation of tumor growth, while increases are associated with tumor progression [35]. In this study, most of the 28 patients with post-treatment LDH that decreased to normal after chemotherapy, had a good response to chemotherapy (PR or CR), and ORR was significantly higher than that in patients with abnormal post-treatment serum LDH higher than baseline. This suggested that LDH changes after chemotherapy reflect tumor regression. However, no significant advantages were found for patients with post-treatment LDH that did not decline to normal and patients with both normal baseline and post-treatment LDH. Likely tumor regression occurred with the rapid and dramatic fall in LDH and this might explain why patients whose post-treatment LDH decreased to normal had longer PFS.

Therefore, serum LDH may be a useful biomarker to predict survival and chemotherapeutic response in advanced TNBC patients. More research is needed to confirm these findings and to understand the mechanism underlying LDH and tumor progression.

## MATERIALS AND METHODS

A retrospective review was undertaken with female patients with advanced TNBC (*n* = 131) who received first-line chemotherapy at Fudan University Shanghai Cancer Center between 2005 and 2013. Patients were included if they had pathological confirmation of TNBC, biopsy or radiological confirmation of tumor recurrence or distant metastasis, good ECOG performance status, and available data on baseline and post-treatment serum LDH levels (see patient characteristics in Table 1). Patients were excluded if they had complications from other types of malignancies, no evidence of tumor recurrence or distant metastasis, acute heart failure, or severe renal or liver dysfunction.

Patients’ medical charts were reviewed to obtain data about clinical features, treatment information, and serum LDH levels. Menopause status was defined according to the National Comprehensive Cancer Network (NCCN) Guidelines (NCCN Clinical practice guidelines in oncology. Breast Cancer V1 2017 [36]). Serum LDH levels were measured using the Roche Cobas 8000 system (Roche, Indianapolis, IN, USA). All patients were told to fast after midnight on the day of the blood test. Baseline serum LDH levels were measured within 2 weeks before administering first-line chemotherapy. If multiple measurements were taken, the average of the measurements was used. Patients were stratified to normal baseline LDH (≤ 250 IU/L) and abnormal baseline LDH (>250 IU/L) groups. Post-treatment serum LDH measurements were conducted after two cycles of first-line chemotherapy. According to changes between baseline and post-treatment LDH values, patients were divided into four groups: Group 1 included patients with abnormal post-treatment serum LDH levels higher than baseline; Group 2 included patients with post-treatment serum LDH levels that decreased but did not return to normal; Group 3 included patients with post-treatment serum LDH levels that decreased to normal; and Group 4 included patients with normal baseline and post-treatment LDH levels. Tumor responses to chemotherapy (chemotherapeutic response) were assessed and graded as complete response (CR), partial response (PR), stable disease (SD), or progressive disease (PD) according to the revised RECIST guideline (version 1.1) [37]. Clinical response was defined as either CR or PR. Survival information was collected from medical records or telephone interviews. The disease-free interval (DFI) was defined as the period between surgical resection and diagnosis of tumor recurrence or distant metastasis. Progression-free survival (PFS) was defined as the period between the start of chemotherapy and the first time of documented PD. Overall survival (OS) was defined as the period between diagnosis of local recurrence or distant metastasis and death or last follow-up. Objective response rate (ORR) was defined as the percent of patients with CR and PR.

We used SPSS 21.0 statistical software to analyze data, and a two-tailed *p* value < 0.05 was considered statistically significant. A multiple comparisons analysis was not mandatory because the goal of this exploratory analysis was to identify hypotheses that could be subject to more rigorous future examinations. A chi-squared test was used to investigate the association between baseline serum LDH levels and clinical features, as well as the association between various risk factors and chemotherapy response. Logistic regression was used to analyze the association between serum LDH levels and potentially relevant factors, as well as the association between the chemotherapeutic response and potential relevant factors. PFS and OS were determined according to the Kaplan–Meier method, and intergroup comparison according to the variables in Table 1 was performed by the log-rank test. Subsequently, the relevant variables identified by univariate analysis with a significant association with PFS and OS (*P* < 0.1) and two clinically potentially important variables (age and type of first-line treatment) were analyzed using the Cox regression model to determine the independent prognostic factors for PFS and OS.
